# “Big men” in the office: The gender-specific influence of weight upon persuasiveness

**DOI:** 10.1371/journal.pone.0222761

**Published:** 2019-11-11

**Authors:** Kevin M. Kniffin, Vicki L. Bogan, David R. Just

**Affiliations:** Dyson School of Applied Economics and Management, SC Johnson College of Business, Cornell University, Ithaca, NY, United States of America; Universita degli Studi di Catania, ITALY

## Abstract

Height has been closely studied as a factor that influences myriad measures of leadership; however, the potential influence of weight on socially beneficial traits has been neglected. Using the anthropological concept of “big men” who relied on influence to lead their communities, we examine the role of weight upon persuasiveness. We present the results of six studies that suggest a tendency for raters to expect larger body mass to correspond with more persuasiveness among men. In the sixth, pre-registered study, we find evidence that fits the hypothesis that weight among men is positively associated with perceived persuasiveness. While the “big man” leadership concept is based on studies of pre-industrial societies where weight embodied status, our findings suggest an evolved bias to favor moderately big men–with respect to perceived persuasiveness–even in environments where there is no reason to interpret over-consumption of food and conservation of energy as a signal of wealth. Our studies contribute novel perspectives on the relevance of weight as an understudied dimension of “big” and offer an important qualification informed by evolutionary perspectives for the stigmatizing effects of relatively large body mass.

## Introduction

Anthropologists studying small-scale pre-industrial societies commonly and traditionally described community leaders–who needed to rely on persuasiveness rather than institutional power or force–as “big men” [1]. Indeed, recent studies of hunter-gatherers have found that physical size tends to have a positive relationship with leadership among men [2,3]. Taken literally, “big” can refer to at least two physical dimensions: height or weight. The dimension of height has been closely studied in relation to leadership styles [4], career progression [5], income [6], decision making [7], and risk taking [8–10]. Research examining the height of United States (US) Presidents–even with a sample size of fewer than 50 males–highlights the direct and indirect influences that people expect to be associated with being tall or short [11,12]. As an illustration of the generally positive relationship that has been found between height and leadership–especially but not exclusively for men [13], Murray and Schmitz [14] report that men who are relatively tall are more likely to view themselves as qualified to serve in leadership positons.

Weight, on the other hand, has been understood to be a sign of elevated status in pre-industrial societies where greater weight reflected a combination of high resources and low energy expenditures [15–17]; however, it is common for studies to show that people in contemporary, post-industrial environments tend to place greatest value on being relatively thin [18,19]. In fact, the notion that people in contemporary environments hold negative stereotypes about obese people has been well-documented [20,21] across an array of interpersonal connections. In clinical contexts, for example, healthcare professionals have been shown to regard overweight patients negatively [22,23]; and, in political theaters, overweight candidates are accustomed to having their weight discussed as part of their candidacies. For example, when New Jersey Governor Chris Christie was considered to be a potential contender for the 2012 Republican Presidential nomination, pundits questioned whether a person with Christie’s physical girth could win [24]. Subsequently, Christie underwent a voluntary surgical procedure designed to help him control his weight, sparking a new round of speculation that he was preparing–as he was–to challenge for a White House bid in 2016 [25]. The implication from these episodes involving Christie’s weight is that people tend to consider obesity to be a barrier to becoming a leader.

When comparing height and weight as dimensions of “bigness,” there are obviously differences between the variables. For example, people can lose weight much more quickly than height [26]. More generally, people often view weight to be affected by individual choice–unlike height–and King et al. [27] highlight that when people assume that others can control their weight, then it becomes easier to justify prejudices against overweight or obese individuals. Notwithstanding these important and basic differences between height and weight, it is notable that prior research has examined the degree to which temporary increases in a person’s size (e.g., via “power posing”) might affect a person in ways that potentially relate to persuasiveness, but such work has reached inconsistent conclusions [28,29]. Similarly, there exists prior work suggesting that a person carrying extra weight temporarily (for an experiment) will tend to perceive and perhaps behave with more assertive orientations [30,31] but recent attempts to replicate some of those findings also have yielded inconsistent conclusions [32–35].

Intermediate between the relatively permanent trait of height (among adults) and the substantially more flexible nature of a person’s posture, it is also notable that prior research has examined the degree to which a person’s physical strength might affect a person’s disposition towards negotiating various types of non-physical conflict [36–38] as well as the degree to which people who appear physically stronger tend to be accorded higher social status [39]. Such research into the relevance of physical formidability is often motivated by evolutionary perspectives and points to an additional dimension of bigness that is outside of the scope of our interests but helpful for contextualizing our focus on weight.

Through this article, we integrate perspectives from multiple disciplines to consider the degree to which weight–the other main dimension of “big”–might favorably contribute to perceptions of persuasiveness, perhaps specifically among men. While popular phrases such as “throwing one’s weight around” or “playing the heavy” imply a positive relationship between weight and persuasiveness, previous research typically has not examined the connection closely. Just as research focusing on height and leadership tends to apply an evolutionary perspective [40], we focus on whether the positive association between weight and leadership, suggested by anthropologists to have been prominent during the “environment of evolutionary adaptedness” [41], has relevance for people in contemporary environments. Whereas weight embodied status in pre-industrial environments because it signaled the ability to consume surplus goods without expending comparable amounts of energy, we examine the degree to which persuasiveness might be favorably influenced by weight notwithstanding the more conventional stigmatized view of being overweight [42–45]. Two recent exceptions to our review of prior research [46,47] examined whether and how a variety of “big” dimensions are correlated with interpersonal perceptions and behavior, both finding that weight does not appear to have an independent influence when compared alongside other variables such as height.

While social scientists from fields including sociology have been “highly suspicious” of evolutionary perspectives during much of the recent past [48], it is notable that our application of evolutionary anthropology is focused on considering a potentially positive aspect of a condition that is most typically stigmatized in contemporary social environments. In other words, while it would be appropriate to be suspicious of anyone claiming that discrimination against overweight people is based on biological or genetic factors, our focus is clearly differentiated from that type of pursuit. In the affirmative, it is worth highlighting that our approach is similar to studies of status striving that have employed evolutionary perspectives [49]. Our approach does not presume that genetics is responsible for individual preferences in relation to weight-perceptions; however, we do adopt the common assumption among evolutionary social scientists that people are not presumed to be consciously aware of evolved preferences (e.g., for “big” leaders). As a result of this assumption, which is certainly found in other broad explanatory frameworks (e.g., Freudian analyses), our investigation into preferences for “bigness” employs an array of different research methods.

### Evolutionary mismatch on weight and status

As with studies of height in relation to non-manual work environments (e.g., being President of the United States), there is no essential relationship that should exist for weight. With the exception of certain athletic occupations such as sumo wrestlers or linemen on professional football teams, there is no clear rationale or consensus for why weight should be relevant for many contemporary occupations. In this respect, our focus examines whether the value that people accord to being big for tasks is mismatched with actual requirements of respective activities. We focus specifically on persuasiveness since “big men” in pre-industrial societies did not have the power of an institution to enforce their standing in the group’s hierarchy; instead, “big men” needed to rely upon influence and persuasiveness to gain and maintain their status.

The question of whether weight is healthy or not is independent from the question of weight’s potential influence on interpersonal interactions. As Campbell [50] wrote about “the conflicts between biological and social evolution” when describing the potential for vestigial traits to exist: “the wisdom produced by evolutionary processes (biological or social) is wisdom about past worlds. If there are grounds for believing that the relevant aspects of those worlds have changed, past adaptations may now be judged to be maladaptive.” In the case of weight, the conventional view from medical researchers is that being overweight or obese tends to predict negative health outcomes [51]; however, that contemporary judgment of weight as maladaptive is fully distinct from whether people continue to accord some favorable traits to being overweight or obese. Our approach to the relationship between being overweight and individual interpersonal patterns partly mirrors DeWall et al.’s [52] finding that “big guys” are more likely to become aggressive when intoxicated. More broadly, our approach to juxtapose a pre-industrial marker of leadership (i.e., weight) in contemporary environments illustrates the value of evolutionary perspectives–recently highlighted by Von Rueden and Van Vugt [53]–for understanding dynamics between leaders and followers.

### Big names and heavyweights

As a pilot study that helped inform our approach to this topic, we asked 78 adult participants recruited from undergraduate business courses from a private university in the Northeastern U.S. in exchange for partial course credit to identify “What are the first three words you imagine when you see the word “heavyweight” or “lightweight.” The most salient contrast that we found is that people often viewed heavyweight to be “strong” whereas lightweight prompted responses that covered an array of socially undesirable traits such as “weak,” “pushover,” and “unqualified.” While our pilot study was intended as exploratory, it is notable that such a contrast fits with our expectation of positive associations involving heaviness.

The other main finding from our freelisting task is that–unlike “big name” or “big star”–people often associate heavyweight and lightweight with boxing since they are common classifications in which boxers compete. It is notable in the context of our focus, though, that heavyweight champions such as Muhammad Ali, Joe Frazier, and Mike Tyson have traditionally drawn significantly greater interest across the world than boxers who weigh less [54]. While it is plausible that the heaviest and biggest boxers might also tend to be the best, the great majority of people have occupations and lives that do not include boxing; consequently, the degree to which attraction to “big” tends to cut across activity domains is an important question to examine since being big would seem to be irrelevant to most contemporary jobs. Notably, it is consistent with the findings of our pilot study that a recent article by evolutionary psychologists [55] found that people tend to be perceive that “bigger is better” with respect to an athlete’s overall size (i.e., inclusive but not limited to considerations of weight).

## Research overview

We conducted a series of six studies with independent samples (with approval from the Cornell University Institutional Review Board and with informed consent provided by participants) to examine the basic nature of weight’s relationship with persuasiveness and perceptions of persuasiveness with a specific interest to examine the Research Question (RQ) whether “big men” are viewed–as would be predicted by an evolutionary perspective–as relatively persuasive when compared with normal weight men. Given that the phrase “big man” lacks a corresponding “big woman” in anthropological studies, one could infer that gender may be a key factor in the relationship between bigness and leading. In fact, the general tendency for obese women to be penalized more than obese men with respect to wages [56] offers a practical line of argument consistent with the anthropological backdrop. Conversely, contemporary phrases such as “big name” or “big star”–referring to very successful performers across activity domains such as arts, sciences, and politics–suggest that any relationship between weight and persuasiveness might be genderless. We also specifically will examine the issue of gender in one of our six studies.

Study 1 considers weight in relation to self-reported persuasiveness. Studies 2 and 3 present participants with genderless stimuli in order to assess any general relationship between perceptions of weight and persuasiveness. Study 4 builds upon the earlier studies with a diverse set of stimuli to focus on whether the persuasiveness of big men and big women is differently perceived; and, Study 5 was conducted with a non-Western sample in order to address concerns regarding research that draws exclusively on Western, Educated, Industrialized, Rich, and Democratic (WEIRD) populations [57]. As a confirmatory test, Study 6 presents a pre-registered test of the main finding generated by Studies 4 and 5.

The basic starting point for our interests draws on Study 1’s consideration of individuals’ own behaviors as a function of their weight. While prior research has examined the effects of being obese and/or perceived as obese on social relationships with others [58], we focus on whether there is a direct relationship between being overweight and persuasive. As further explorations, our subsequent studies increasingly focus on the nature and degree to which people perceive weight and persuasiveness to be positively related. Studies 2 and 3 are designed partly as projection tests to help avoid response biases given that people are not likely to self-report their own discrimination of others based on others’ weight. As complements, Studies 4–6 are based on direct ratings that participants provided in response to variably-weighted images. Unlike Studies 2 and 3, we also designed Study 4 to measure the degree to which preferences for “bigness” might be gender-specific (e.g., to “big men”) and Studies 5 and 5 to focus on how variably sized men are differentially perceived. Taken together, the set of studies offers multiple perspectives on the various ways in which weight, persuasiveness, and gender appear interrelated in ways that differ from research that looks more bluntly at an overall stigma associated with being overweight.

## Study 1: Persuasiveness and weight

### Method

#### Participants

One hundred and forty-one undergraduate students (81 women and 60 men) from a private university in the Northeastern U.S. were recruited from undergraduate business classes and participated in exchange for compensation.

#### Procedures

We asked participants to complete the persuasion subscale of the Leader Behavior Description Questionnaire (LBDQ) [59,60], which asks people to rate the frequency with which they engage in a set of 10 behaviors (e.g., being a convincing speaker) on a 5-point scale ranging from Always to Never (α = 0.90). Subsequent to their completion of the LBDQ, participants also reported their weight and height.

### Results and discussion

Correlational analyses showed a significant positive relationship between participants’ self-reported weight and persuasiveness (*r* = 0.17, *p* = 0.04) across the full sample. With respect to other important variables, persuasiveness did not correlate significantly with gender (*r* = 0.13, *p* = 0.12); however, the trait did significantly correlate with height (*r* = 0.20, *p* = 0.02).

Regression analyses that incorporated height and gender along with weight showed that weight did not have an independent relationship with persuasiveness. Given possible concerns about multicollinearity, we calculated variance inflation factors (VIFs) for the regression models, which included height and gender alongside weight as independent variables, and found acceptable values [61] of no more than 2.42. While the regression results suggest that height underlies the correlation between weight and persuasiveness, researchers who focus on obesity are clear to note that the relationship between weight and height is complex and imperfectly understood [62]. For example, individuals sharing the same height and weight can also have substantially different percentages of muscle, body fat, and weight circumstance.

Study 1, consequently, provides a very limited degree of support–through the bivariate correlation involving weight–for the “big man” model of persuasiveness; however, our analyses are also clear to highlight that “bigness”–as a function of height, weight, and overall body shape–requires finer grained tests. Given that the basic correlational finding involving weight and persuasiveness invites closer investigation and given gaps in our understanding of the relationship between weight and height [62], we conducted studies 2 through 6 in order to focus more closely on the relevance of weight by controlling more directly for variations in height.

## Study 2: Persuasiveness and the biases of others

### Method

#### Participants

Forty-two undergraduate students (27 women and 15 men) from a private university in the Northeastern U.S. were recruited from undergraduate business classes and participated in exchange for partial course credit.

#### Procedures

Using a within-subjects design, we asked participants to rate their level of disagreement or agreement on a 9-point scale (with 1 being the highest level of disagreement and 9 being the highest level of agreement) for the following four statements: “I think that heavy people are more likely to be persuasive,” “I think that heavy people are more likely to be perceived as persuasive,” “I think that light people are more likely to be persuasive,” and “I think that light people are more likely to be perceived as persuasive.”

### Results and discussion

As suggested by the descriptive statistics in Table 1, participants agreed most strongly with the statement that “heavy people are more likely to be perceived as persuasive.” Paired contrasts between the heavy and light variants are not significantly different; however, there is a significant difference between how raters personally estimated the persuasiveness of heavy people when compared with how they expect others to regard heavy people (*t* = -2.75, *p* < 0.01, Cohen’s *d* = 0.43 corrected according to Morris & DeShon [63]). A comparison of ratings based on the rater’s gender showed no significant differences.

**Table 1:** Descriptive statistics for study 2 (n = 42).

Agreement Ratings for Self (1 = strongly disagree; 9 = strongly agree)	Mean	S.D.	Agreement Ratings for Self (1 = strongly disagree; 9 = strongly agree)	Mean	S.D.
I think that heavy people are more likely to be persuasive	4.31	1.85	I think that heavy people are more likely to be perceived as persuasive	4.86	1.84
I think that light people are more likely to be persuasive	4.43	2.05	I think that light people are more likely to be perceived as persuasive	4.52	2.12

The interesting finding from these results is that people expect others to show a favorable bias for weight in relation to persuasiveness; however, they do not personally report such a bias. One interpretation of this pattern is that participants were influenced by a conscious response bias [64] to avoid self-reporting prejudices in relation to weight. In order to address this possibility, we employed between-subject designs in Study 3 so that participants would not directly consider the influence of relative weight upon their perceptions.

## Study 3: Projection test of gravitas

### Method

#### Participants

One hundred and thirty-one adults (46 women and 85 men) from the U.S. were recruited from a validated online sample [65] and participated in exchange for monetary compensation.

#### Procedures

Using a between-subjects design, participants were randomly presented with one of two prompts: “Given that ‘Gravitas’ is a word that is sometimes used to describe persuasive political or corporate leaders, on the scale of relative weight, please indicate how a person WITH (or WITHOUT) Gravitas is likely to look.” Participants were then asked to estimate on a four-point scale whether the person would be (1) Underweight, (2) Normal Weight, (3) Overweight, or (4) Obese. Subsequent to their completion of this task, we also asked participants to indicate whether they had known the meaning of the word “gravitas” prior to the study.

### Results and discussion

**Fig 1:**
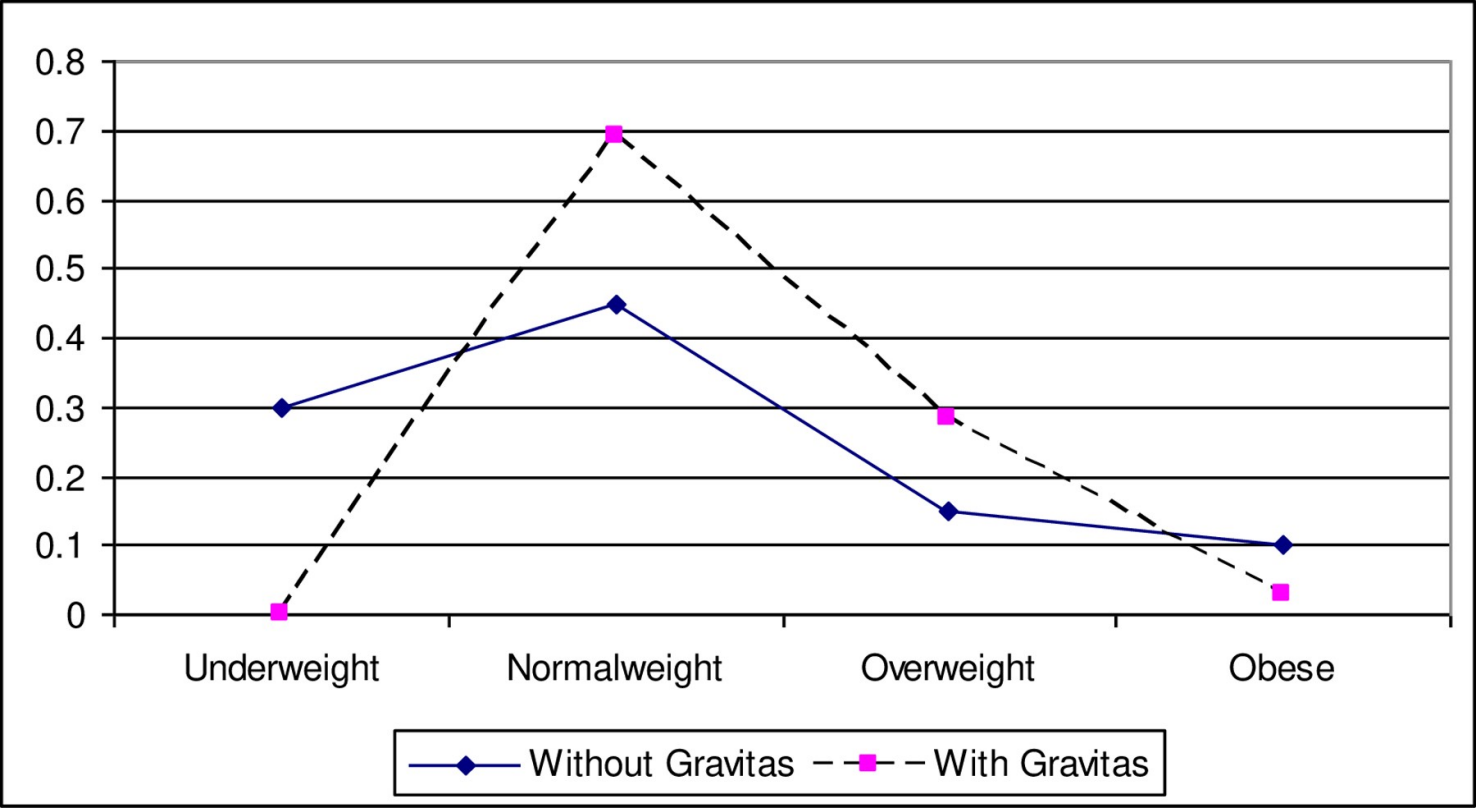
Relative distribution of expected weight categories as a function of “Gravitas”.

For the full sample, Fig 1 illustrates a significant difference whereby participants estimated that a person with gravitas would weigh more. More specifically, an analysis of variance shows that raters estimated someone with gravitas to be above normal weight (M = 2.34, SD = 0.53) and a person without gravitas to be close to normal weight (M = 2.05, SD = 0.93) (*F* = 4.92, *p* = 0.03; Cohen’s *d* = 0.38). A regression for raters’ prediction of weight that considered the influence of the rater’s gender showed a uniquely significant influence of condition (with or without gravitas) (*B* = 0.29, *t* = 2.21, *p* = 0.03) and null effect for rater’s gender (*B* = 0.01, *t* = 0.09, *p* = 0.93). A regression model that included an indicator variable for whether a participant had prior knowledge of the word gravitas showed an identical pattern whereby the experimental gravitas condition mattered (*B* = 0.35, *t* = 2.61, *p* = 0.01) while prior knowledge of the word did not (*B* = 0.09, *t* = 0.64, *p* = 0.52) for estimating weight based on the concept.

Given (a) the substantial number of participants (55%) who self-reported that they did not know the meaning of the word “gravitas” prior to the study and (b) the possibility that additional participants overstated their knowledge of the word, Study 3 functions partly as a projection test that assesses how much participants expect persuasive and unpersuasive people to weigh. While Study 2’s within-subject design required participants to directly consider the possibility that weight influences their perceptions of others’ persuasiveness, Study 3 demonstrates a robust bias for people to expect persuasive people to weigh more than non-persuasive people. It is interesting in this regard that prior work has found that people consider relatively heavier objects to be relatively more important [30,66]; however, our approach to considering the relevance of heaviness among people–in relation to persuasiveness–offers a novel perspective.

## Study 4: Line drawings of men and women

### Method

#### Participants

One hundred and sixteen adults (43 women and 73 men) from the U.S. were recruited from a validated online sample and participated in exchange for monetary compensation.

#### Procedures

We presented participants with a set of line drawings (available from the corresponding author) that varied as a function of weight. Starting with the drawings that Singh [67,68] developed, which have been standard illustrations of various weight categories within the field of evolutionary psychology, we adapted the under-, normal-, and over-weight images for (a) women with a waist-to-hip ratio (WHR) of 0.7 and (b) men with a WHR of 1.0 and we added proportionately-larger obese drawings to each series. In addition to controlling for WHR within the series of variably-weighted images for women and men, our approach utilized the WHRs that are typically considered most attractive [67,68]. We can add that–while there exists a wider range of body shapes that could be shown to characterize relatively large body mass and, more specifically, while people with relatively high BMIs can certainly also have high levels of physical fitness [69–71]–our identification of the overweight and obese images conforms to conventional labeling that is based on body mass index (BMI), as used by the Center for Disease Control (CDC) (https://www.cdc.gov/healthyweight/assessing/bmi/adult_bmi/index.html).

Participants were presented with the series of male or female drawings–without any labeling in relation to weight–in random order and asked to rate each image in the series on a 9-point scale with respect to how persuasive, extraverted, humorous, and physically attractive they expected the person to be. We included several other variables for participants to rate in order to avoid holistic ratings for our focal variable of persuasiveness. More specifically, we included the set of three additional variables given prior research showing relationships with both perceptions of weight as well as workplace interests. For example, Cowart and Brady [72] review the relationships between obesity and perceived joviality in the context of how frontline service employees are perceived by customers. Similarly, Kniffin et al. [73,74] review the relevance of physical attractiveness for workplace dynamics.

### Results and discussion

**Fig 2:**
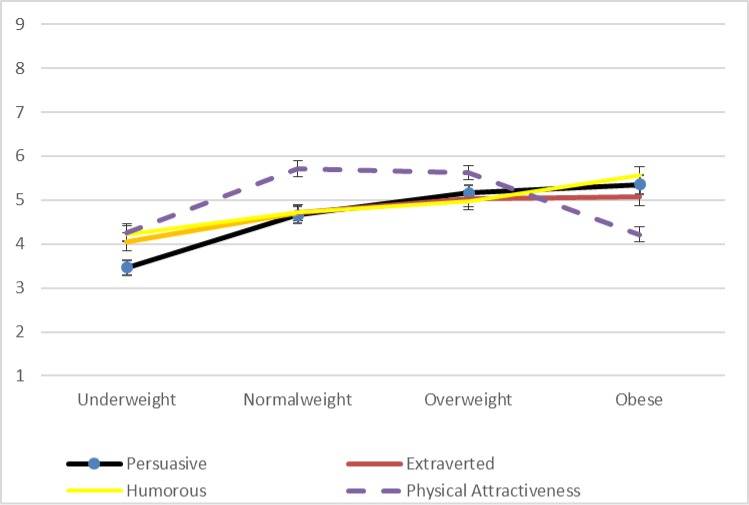
Mean ratings for male line drawings for study 4 (Error bars represent the standard error [SE] for each value).

**Fig 3:**
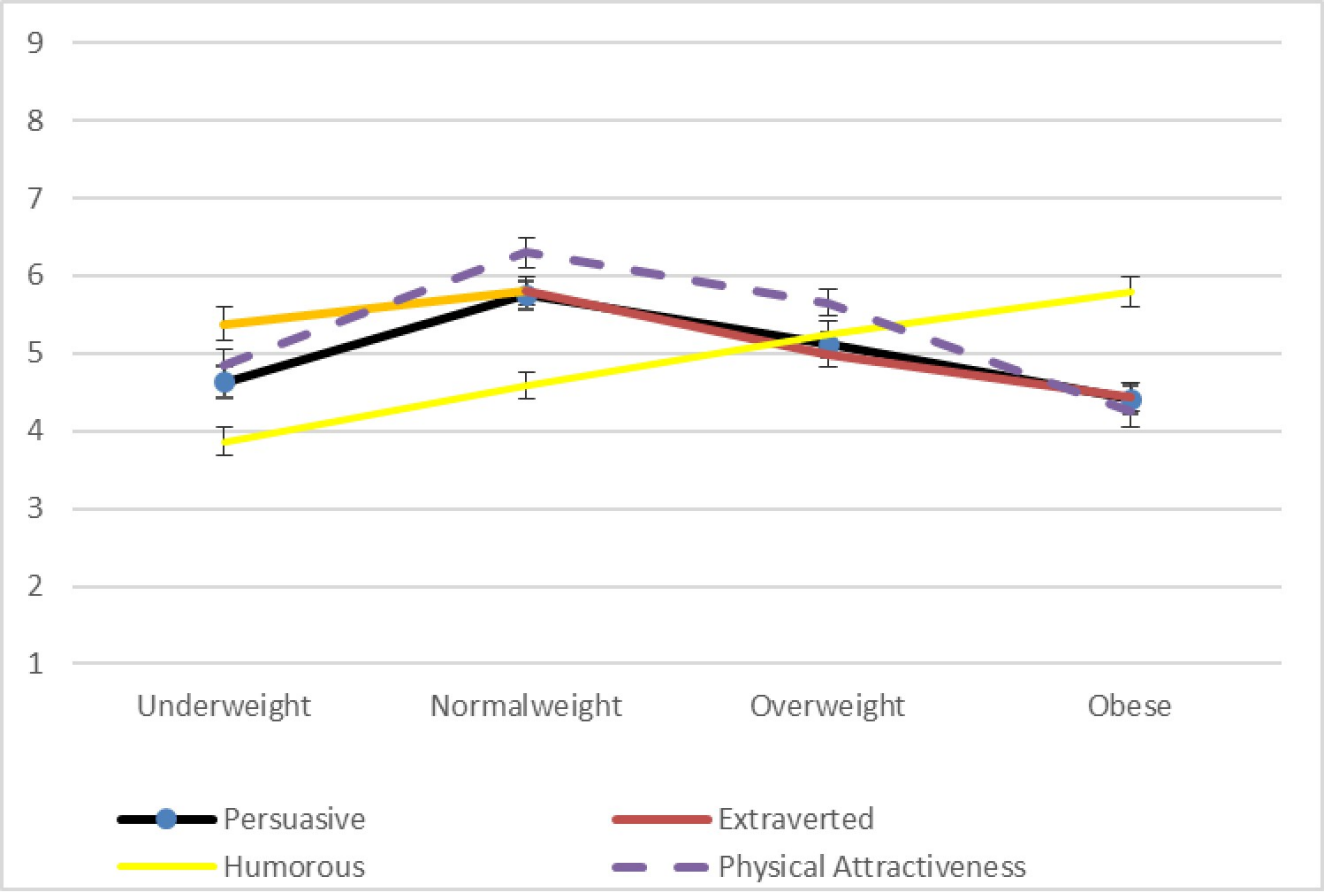
Mean ratings for female line drawings for study 4 (Error bars represent the standard error [SE] for each value).

Focusing initially on ratings of persuasiveness for the male and female drawings independently, significant differences for the male drawings’ persuasiveness ratings exist according to an analysis of variance across the four weight categories (*F* = 21.0, *p* < 0.01). In closer detail, the bivariate correlation values between persuasiveness ratings for the male line drawings and the four indicator variables for underweight (*r* = -0.33, *p* < 0.01), normal weight (*r* = 0.00, *p* = 0.93), overweight (*r* = .13, *p* < 0.01), and obese (*r* = .19, *p* < 0.01) suggest a positive relationship between weight and persuasiveness. This trend is visible in Fig 2, which shows the ratings for all four variables for the male line drawings. In contrast, as visible in Fig 3, there is no linear trend among the persuasiveness ratings for the female line drawings.

Table 2 reports the aggregated descriptive statistics and correlations for the full sample, inclusive of the ratings assigned to the male and female line drawings. More specifically, Table 2 draws on the eight ratings that each participant provided for the two sets of four male and female line drawings.

**Table 2:** Study 4 descriptive statistics and correlations (n = 116 participants [928 observations]).

Variables	*M*	*S*. *D*.	1	2	3	4	5	6	7	8	9
**1. Under weight stimulus (0/1)**	0.25	0.43									
**2. Normal weight stimulus (0/1)**	0.25	0.43	-0.33**								
**3. Over weight stimulus (0/1)**	0.25	0.43	-0.33**	-0.33**							
**4. Obese stimulus (0/1)**	0.25	0.43	-0.33**	-0.33**	-0.33**						
**5. Persuasiveness (1–9)**	4.82	2.13	-0.22**	0.11**	0.09**	0.02					
**6. Extraversion** **(1–9)**	4.93	2.10	-0.06*	0.09**	0.02	-0.05	0.44**				
**7. Humor (1–9)**	4.87	2.07	-0.23**	-0.06	0.07*	0.23**	0.35**	0.28**			
**8. Physical Attractiveness (1–9)**	5.11	2.17	-0.15**	0.24**	0.14**	-0.23**	0.48**	0.38**	0.26**		
**9. Female Rater (0/1)**	0.37	0.48	-0.01	0.01	-0.01	-0.01	0.05	-0.03	0.03	-0.05	
**10. Male Drawing (0/1)**	0.50	0.50	0.00	0.00	0.00	0.00	-0.07	-0.10*	-0.00	-0.07*	0.00

* p < 0.05

** *p* < 0.01

In order to examine the degree to which the other three traits that we measured might have influenced the patterns in Fig 2 and Table 3 presents the results of a regression analysis for ratings of persuasiveness for the male line drawings when controlling for ratings of extraversion, humor, and physical attractiveness. To control for the non-independence of four ratings from each rater, we used a cluster-robust variance estimator [75]. Generally, Table 3 indicates that–when compared with the reference set of normal weight individuals–persuasiveness ratings are significantly higher (*p* < 0.01) for ratings of the obese drawing and are nominally higher (*p* = 0.06) for ratings of the overweight drawing. Persuasiveness is also significantly lower (*p* < 0.01) for underweight images compared with the normal standard.

**Table 3:** Influence of weight upon perceived persuasiveness of male line drawings in study 4 (Normal weight is reference weight) (n = 116).

Variable	Coefficient	S.E.	*z*	*p*
**Underweight**	-0.48	0.18	-2.60	**0.01**
**Overweight**	0.43	0.21	2.00	0.06
**Obese**	1.04	0.30	3.50	**0.00**
**Extraverted**	0.20	0.07	2.88	**0.01**
**Humorous**	0.13	0.07	1.80	0.07
**Physically Attractive**	0.36	0.08	4.70	**0.00**
**Female Rater**	0.14	0.17	0.87	0.35
**Constant**	0.94	0.40	2.36	**0.02**

With respect to the other variables presented in Table 3, participants who rated drawings as more physically attractive also tended to provide significantly higher persuasiveness scores (*p* < 0.01) just as those drawings that were rated as more extraverted were also rated as significantly more persuasive (*p* = 0.01). Notably, though, with respect to potential concerns about multicollinearity, the average VIFs for the group of our independent variables is well within acceptable limits, ranging from 1.11 to 2.89 with an average of 1.94. In the affirmative, we can also observe that the effect sizes (coefficients) in Table 3 show that the weight conditions (i.e., obesity) have the largest influence in the model. Finally, Table 3 also indicates no significant difference between ratings provided by male and female raters.

While the other three variables that we measured are not the focus of our interest, we can note that ratings for the female line drawings do show a significant linear trend with respect to ratings of expected humor (*F* = 21.3, *p* < 0.01). In contrast, as is visible in Fig 3, ratings for extraversion and physical attractiveness–as well as persuasiveness–peak for normal weight before declining linearly in relation to overweight and obese. For the male line drawings, extraversion and humor varied significantly within subjects and followed the same linear trend as persuasiveness (see Fig 2). With respect to male physical attractiveness, Table 3 suggests that the gains in persuasiveness, extraversion, and humor are balanced against a low rating for physical attractiveness.

## Study 5: Replication of study 4 findings in non-western sample

In order to assess the robustness of the significant linear trend found in Study 4 with respect to ratings of persuasiveness among the male line drawings in addition to addressing concerns regarding research that relies exclusively on sampling in WEIRD populations [57], we conducted Study 5.

### Method

#### Participants

227 adults (98 women, 127 men, and 2 respondents who did not indicate male or female) from Kenya with fluency in English were recruited by Qualtrics and participated in exchange for monetary compensation.

#### Procedures

Participants were administered the same protocol as Study 4 with the exception that ratings were requested exclusively for male line drawings.

### Results and discussion

**Fig 4:**
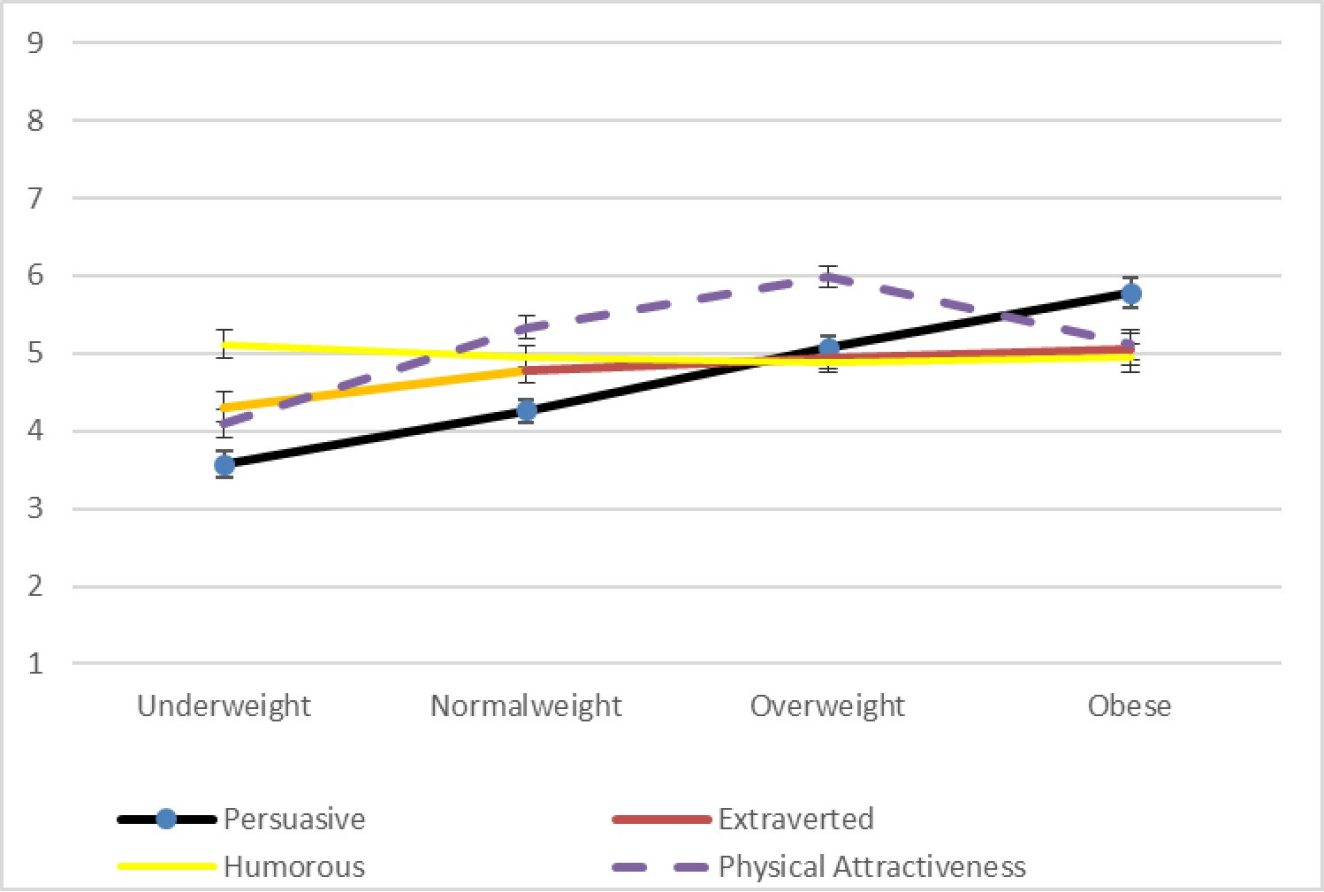
Mean ratings for male line drawings for study 5 (Error bars represent the standard error [SE] for each value).

Focusing on participants’ ratings of persuasiveness as the main variable of interest, we can note that significant differences for the male drawings’ persuasiveness ratings exist according to an analysis of variance across the four weight categories (*F* = 34.1, *p* < 0.01). In closer detail, as visible in Table 4, the bivariate correlation values between persuasiveness ratings for the male line drawings and the four indicator variables for underweight (*r* = -0.24, *p* < 0.01), normal weight (*r* = -0.09, *p* < 0.01), overweight (*r* = 0.09, *p* < 0.01), and obese (*r* = .25, *p* < 0.01) suggest a positive relationship between weight and persuasiveness. This trend is visible in Fig 4, which shows the ratings for all four variables for the male line drawings.

**Table 4:** Study 5 descriptive statistics and correlations (n = 227 participants [908 observations]).

Variables	*M*	*S*. *D*.	1	2	3	4	5	6	7	8
**1. Under weight stimulus (0/1)**	0.25	0.43								
**2. Normal weight stimulus (0/1)**	0.25	0.43	-0.33**							
**3. Over weight stimulus (0/1)**	0.25	0.43	-0.33**	-0.33**						
**4. Obese stimulus (0/1)**	0.25	0.43	-0.33**	-0.33**	-0.33**					
**5. Persuasiveness (1–9)**	4.67	2.62	-0.24**	-0.09**	0.09**	0.25**				
**6. Extraversion** **(1–9)**	4.77	2.59	-0.10**	-0.00	0.04	0.06	0.32**			
**7. Humor (1–9)**	4.98	2.49	0.03	-0.00	-0.02	-0.01	0.14**	0.26**		
**8. Physical Attractiveness (1–9)**	5.13	2.59	-0.23**	0.05**	0.19**	-0.00	0.29**	0.23**	0.09*	
**9. Female Rater (0/1)**^**1**^ (2 missing values)	0.44	0.50	-0.00	-0.00	-0.00	-0.00	0.04	0.07	0.03	0.06

* p < 0.05

** *p* < 0.01

More specifically, Table 5 shows additional evidence of the linear positive relationship between persuasiveness ratings and body mass for the male line drawings even when taking into account the other three variables that were measured as well as whether the rater was a woman.

**Table 5:** Influence of weight upon perceived persuasiveness of male line drawings in study 5. (Normal Weight is Reference Weight) (n = 227).

Variable	Coefficient	S.E.	*t*	*p*
**Underweight**	-0.35	0.16	-2.17	**0.03**
**Overweight**	0.68	0.18	3.68	**0.00**
**Obese**	1.52	0.26	5.89	**0.00**
**Extraverted**	0.23	0.06	3.90	**0.00**
**Humorous**	0.07	0.06	1.29	0.20
**Physically Attractive**	0.19	0.05	3.68	**0.00**
**Female Rater**	0.07	0.16	0.44	0.66
**Constant**	1.73	0.38	4.50	**0.00**

The substantial similarity in patterns visible through Studies 4 and 5 offers a valuable measure of the robustness of findings with respect to persuasiveness. The replication that Study 5 provides is especially informative since the findings are based on a non-Western sample of participants.

## Study 6: Pre-registered replication of study 4 findings for men

As a further test of the robustness of findings present in Studies 4 and 5, Study 6 provides a pre-registered replication that allows us to test patterns that our previous exploratory studies were able to discover.

### Method

#### Participants

220 adults (76 women and 144 men) from the U.S. were recruited from a validated online sample and participated in exchange for monetary compensation.

#### Procedures

As a replicate for the key part of Study 4 on which we focused in Study 5, we pre-registered and employed identical methods to those in Study 5. In this vein, beyond addressing the RQ that was the focus for Studies 1–5, Study 6 allows us to test the Hypothesis that heavier men will tend to be perceived as more persuasive than lighter men. With materials available at osf.io/7rz4s and osf.io/x83az that stipulate the sampling, measurement, and analytic plans that we established prior to completing Study 6, the replication entailed by Study 6 is designed to directly address questions of research reproducibility that have emerged broadly across all of the sciences in recent years [76].

## Results and discussion

Table 6 reports the descriptive statistics and bivariate correlations for Study 6. As indicated in Table 5, the patterns reported in Tables 2 and 5 are replicated. For example, as visible in Table 6, the bivariate correlation values for Study 6 between persuasiveness ratings for the male line drawings and the four indicator variables for underweight (*r* = -0.36, *p* < 0.01), normal weight (*r* = 0.04, *p* = 0.26), overweight (*r* = 0.13, *p* < 0.01), and obese (*r* = .20, *p* < 0.01) suggest a positive relationship between weight and persuasiveness.

**Table 6:** Study 6 descriptive statistics and correlations (n = 220 participants [880 observations]).

Variables	*M*	*S*. *D*.	1	2	3	4	5	6	7	8
**1. Under weight stimulus (0/1)**	0.25	0.43								
**2. Normal weight stimulus (0/1)**	0.25	0.43	-0.33**							
**3. Over weight stimulus (0/1)**	0.25	0.43	-0.33**	-0.33**						
**4. Obese stimulus (0/1)**	0.25	0.43	-0.33**	-0.33**	-0.33**					
**5. Persuasiveness (1–9)**	4.54	2.41	-0.36**	0.04	0.13**	0.20**				
**6. Extraversion** **(1–9)**	4.49	2.34	-0.21**	0.06	0.09**	0.05	0.53**			
**7. Humor (1–9)**	4.68	2.19	-0.06	0.01	0.02	0.02	0.18**	0.29**		
**8. Physical Attractiveness (1–9)**	4.82	2.27	-0.19**	0.27**	0.11**	-0.19**	0.40**	0.45**	0.26**	
**9. Female Rater (0/1)**	0.35	0.48	-0.00	0.00	0.00	0.00	-0.04	-0.04	-0.06	0.02

* p < .05

** *p* < .01

Rather than falsifying the hypothesis tested, that there exists a positive relationship between weight and perceptions of persuasiveness for men, Table 7 shows a nominally stronger pattern in relation to the hypothesis than we found when first exploring this relationship in Study 4. Also, similar to findings from Studies 4 and 5, the VIF for Table 6 is well within acceptable limits, ranging from 1.02 to 1.95 with an average of 1.56.

**Table 7:** Influence of weight upon perceived persuasiveness of male line drawings in study 6 (n = 220 [880 observations]). (Normal Weight is Reference Weight).

Variable	Coefficient	S.E.	*t*	*P*
**Underweight**	-0.80	0.15	-5.22	**0.00**
**Overweight**	0.48	0.18	2.61	**0.01**
**Obese**	1.15	0.22	5.33	**0.00**
**Extraverted**	0.39	0.04	8.57	**0.00**
**Humorous**	-0.01	0.05	-0.17	0.87
**Physically Attractive**	0.25	0.05	5.32	**0.00**
**Female Rater**	-0.17	0.12	-1.44	0.15
**Constant**	1.48	0.30	5.00	**0.00**

**Fig 5:**
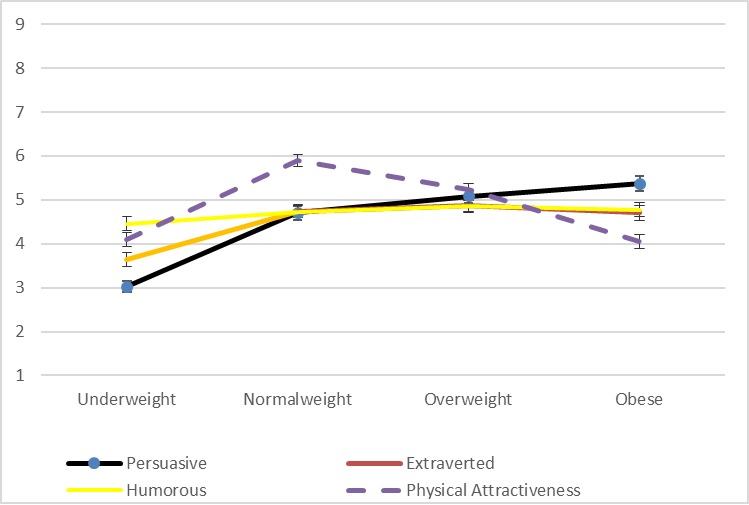
Mean ratings for male line drawings for study 6 (Error bars represent the standard error [SE] for each value).

While the other three variables that we measured are not the focus of our interest, we can note–as is visible in Fig 5 –that there is no comparable linear relationship with weight in any of the cases. Extraversion and humor are lower for underweight drawings and effectively equivalent for the other three while physical attractiveness is highest for normal weight drawings and relatively low for both underweight and obese drawings. In these respects, as with the findings related to persuasiveness, the patterns generated by Study 6 are comparable to those reported by Studies 4 and 5.

## General discussion

While adverse forms of discrimination against overweight and obese individuals in higher socioeconomic environments is commonly and importantly discussed [20,21,77], our studies examine the degree to which weight–like height–might favorably contribute to a person’s perceived persuasiveness. Our studies provide convergent lines of evidence that suggest that people tend to view extra weight or size, at least, to be a positive contribution for men but not women. Against the backdrop of studies showing discrimination against overweight or obese individuals–especially women with above-average body mass index [78,79], our studies suggest that the perception of influential leadership (as measured by persuasiveness ratings) tends to be associated with above-normal weight or size, for men, complementary with prior research showing the benefits of relative tallness for men [11,12].

### Theoretical and practical implications

Motivated partly by anthropological descriptions of “big men” as well as psychological studies of height, our investigations into the role of weight in relation to perceived persuasiveness contribute to general debates concerning the importance of the “environment of evolutionary adaptedness” for life and work in contemporary environments. In somewhat analogous fashion to evolutionary perspectives on the role of height in relation to leadership [40] as well as the importance of strength or formidability for interpersonal ties that do not require strength or formidability [80], our finding of a positive relationship between weight and persuasiveness, whereby big men are perceived as “big” in relation to their persuasiveness, helps to identify a novel trait for which evolved preferences and biases appear to be important. Our focus on weight as a cue for perceived persuasiveness complements previous work [81–84] that has sought to identify facial cues that elicit better-than-random perceptions with respect to leadership-related traits.

To the extent that our studies highlight biases in relation to persuasiveness–a trait that is upstream to broader conceptualizations of leadership, there are clearly practical implications for questions involving negotiations as well as various selection processes. Most generally, while weight–and height–have obvious relevance for work that involves physical activity, there is no clear reason why weight *per se* should directly impact the performance of people engaged in non-manual labor such as political service (e.g., Governor or President). In that respect, employers should be particularly sensitive to the varied ways in which weight colors their perceptions of people with regard to hiring and promotion decisions.

### Limitations and future directions

Our article focuses specifically on persuasiveness rather than a broader array of socially beneficial traits. While this limits the generalizability of our findings for discussions of leadership, our findings invite closer study of the influence of weight in relation to other traits connected with leading, such as assertiveness and charisma. Most immediately, our main focus on perceptions of persuasiveness could be complemented by studies that measure the influence of weight upon actual persuasiveness in groups. For example, just as Campbell et al. [85] find that high waist-to-hip (WHR) ratios among men tends to be associated with the assumption of leadership in randomly assigned workgroups, our findings indicate that the role of weight deserves closer study. Notably, though the stimuli that we presented in Studies 4–6 did not find such a pattern, we expect that a curvilinear relationship exists between weight and persuasiveness for men since–at larger degrees of weight than our stimuli presented–it seems likely that there is some point at which excessive weight will be viewed as universally uninfluential. Such a curvilinear pattern would match prior findings with respect to height whereby very tall men tend to have reproductive fitness that is comparable to relatively short men [86].

Future research that attempts to replicate our findings in other domains–such as corporate executives–could also explore a broader set of variables for which weight might be popularly viewed as an asset for men and/or women. For example, Study 4 shows that heavier women tend to be expected *ceteris paribus* to have a better sense of humor. Beyond expanding the parameter space of perceptions influenced by weight, it would be valuable to assess the relative importance that people ascribe–positively and negatively–to estimates such as persuasiveness and various measures of health. In the case of potential Presidential candidates, for example, people might be more interested to vote for candidates who they expect will have good and stable health when compared with candidates who they expect to be more persuasive. Indeed, criticisms that a President of the United States would substantially under-report his own weight while perhaps over-estimating his own height [87] are interesting in light of the studies we present. Moving beyond perceived or expected relationships involving weight, it would also be valuable to revisit the more direct behavioral relationships that Study 1 considered.

Regarding the stimuli that we used, while the design of Studies 4–6 anticipate possible confounds between weight and physical attractiveness (as highlighted by Nickson et al. [79]), future research will be needed that more clearly disentangles the degree to which perceived differences in strength might have influenced participant ratings. The stimulus-set that we used in Studies 4–6 does follow the conventional depictions of under-, normal-, and over-weight developed by Singh [73,74] and clearly controls for height; however, it is plausible that more varied stimuli (e.g., with different body shapes such as the 3-D images explored by Hu et al. [88]) could generate different patterns of ratings. Indeed, this is sensible in light of the fact that there remain substantial gaps in how the relationships between height and weight are understood [62] along with our own expectation that the positive relationship that we find in Studies 4–6 between height and perceived persuasiveness must eventually have an upper limit after which the relationship would be curvilinear. Further, we can observe that the null finding that we report in Study 1 when height is considered alongside weight offers additional pause for over-interpreting the findings of our current work until finer-grain studies are conducted.

Similarly, while Study 5’s engagement of a non-Western sample offers an important step in response to concerns that research over-relies on WEIRD samples (57), it would be valuable to examine the degree to which patterns reported in our studies are visible across a wide range of cultural groups (e.g., just as Sorokowski [89] has examined the relevance of height in relation to leadership among Presidents of Poland instead of the more commonly studied United States). In these respects, our studies provide baselines for future research into the relevance of weight in relation to other dimensions of physical size as a correlate for leadership-related skills such as persuasiveness.

Lastly regarding broader interpretations, while it is the case that our work was motivated by the “big man” concept developed by evolutionary anthropologists, alternative explanations should be acknowledged. Alternative explanations could be rooted in non-evolutionary or social constructionist perspectives that presume the associations, in this case, between perceived persuasiveness and excess weight among men are learned through cultural experiences (without any vestigial or evolved influences). In this respect, our broader framework for considering perceptions of weight and persuasiveness should be considered against the backdrop of broader criticisms levied by those who question the relevance of evolutionary perspectives for understanding contemporary life [48]. It is also in this context, though, that our engagement of a non-WEIRD sample in Study 5 offers evidence that would challenge a strict social constructionist approach that presumes the paramount importance of cultural learning.

## Conclusion

In contrast with research that highlights the stigma that is commonly associated with being overweight, we present a set of six studies in which we find that the anthropological concept of “big men” can carry literal meaning–in relation to “big”ness and “men”–in contemporary settings. Our studies demonstrate the value of interdisciplinary approaches celebrated by Wood and Ridgeway [90] and show that extra weight is not an asset for women in relation to persuasiveness. In that regard, our detection of opposite biases for men and women affirms the conventional focus on weight’s potential for stigmatizing people.

Prior research has closely examined (i) the importance of height for interpersonal perceptions and (ii) the relevance of weight for health and negative interpersonal relationships; however, our findings open a new line of investigation into the (iii) the influence of weight for positive interpersonal perceptions (e.g., for socially valuable traits such as persuasiveness). Most generally, the suggestion that weight embodies status among men in relation to persuasiveness even in environments where there is no reason to interpret overconsumption of food and conservation of energy as a signal of wealth is intriguing to consider alongside biases that appear to persist vestigially [91] from the distant past into the present. Our findings do not suggest that men should acquire more weight to be viewed as more persuasive; however, our studies do invite closer recognition of benefits that might accrue alongside costs when people carry above-normal weight.

## Supporting information

S1 File Excel file contains worksheets that correspond with each of the article’s studies. (XLS)
